# Temperature-mediated flower size plasticity in Arabidopsis

**DOI:** 10.1016/j.isci.2022.105411

**Published:** 2022-10-21

**Authors:** Andrew Wiszniewski, Estefanía Uberegui, Michaela Messer, Gulmairam Sultanova, Monica Borghi, Gustavo Turqueto Duarte, Rubén Vicente, Katelyn Sageman-Furnas, Alisdair R. Fernie, Zoran Nikoloski, Roosa A.E. Laitinen

**Affiliations:** 1 Molecular Mechanisms of Plant Adaptation Group, Max-Planck-Institute of Molecular Plant Physiology, Potsdam, Germany; 2 Central Metabolism Group, Max-Planck-Institute of Molecular Plant Physiology, Potsdam, Germany; 3 Utah State University, Department of Biology, Logan, UT, USA; 4 Belgian Nuclear Research Centre (SCK CEN), Unit for Biosphere Impact Studies, Boeretang 200 - 2400 Mol, Belgium; 5 System Regulation Group, Max-Planck-Institute of Molecular Plant Physiology, Potsdam, Germany; 6 Plant Ecophysiology and Metabolism Lab, ITQB Nova - Plant Sciences Division, Av. da República, 2780-157 Oeiras, Portugal; 7 Department of Biology, Duke University, Durham, NC 27708, USA; 8 Systems Biology and Mathematical Modelling Group, Max-Planck-Institute of Molecular Plant Physiology, Potsdam, Germany; 9 Bioinformatics, Institute of Biochemistry and Biology, University of Potsdam, Potsdam, Germany; 10 Organismal and Evolutionary Research Programme, Faculty of Biological and Environmental Sciences, Viikki Plant Science Centre, University of Helsinki, Helsinki, Finland

**Keywords:** Plant biology, Plant morphology, Plant genetics, Plant physiology

## Abstract

Organisms can rapidly mitigate the effects of environmental changes by changing their phenotypes, known as phenotypic plasticity. Yet, little is known about the temperature-mediated plasticity of traits that are directly linked to plant fitness such as flower size. We discovered substantial genetic variation in flower size plasticity to temperature both among selfing *Arabidopsis thaliana* and outcrossing *A*. *arenosa* individuals collected from a natural growth habitat. Genetic analysis using a panel of 290 *A*. *thaliana* accession and mutant lines revealed that *MADS AFFECTING FLOWERING* (*MAF*) *2–5* gene cluster, previously shown to regulate temperature-mediated flowering time, was associated to the flower size plasticity to temperature. Furthermore, our findings pointed that the control of plasticity differs from control of the trait itself. Altogether, our study advances the understanding of genetic and molecular factors underlying plasticity on fundamental fitness traits, such as flower size, in response to future climate scenarios.

## Highlights

• Flower size plasticity to temperature in *A*. *thaliana* and *A*. *arenosa* has genetic basis
• The flowers are smaller at higher temperature in most of the *A*. *thaliana* accessions
• *MAF2-5* gene cluster is responsible for flower size plasticity to temperature
• Flower size plasticity is not genetically linked to flower size, as a focal trait

## Graphical abstract

**Figure:**
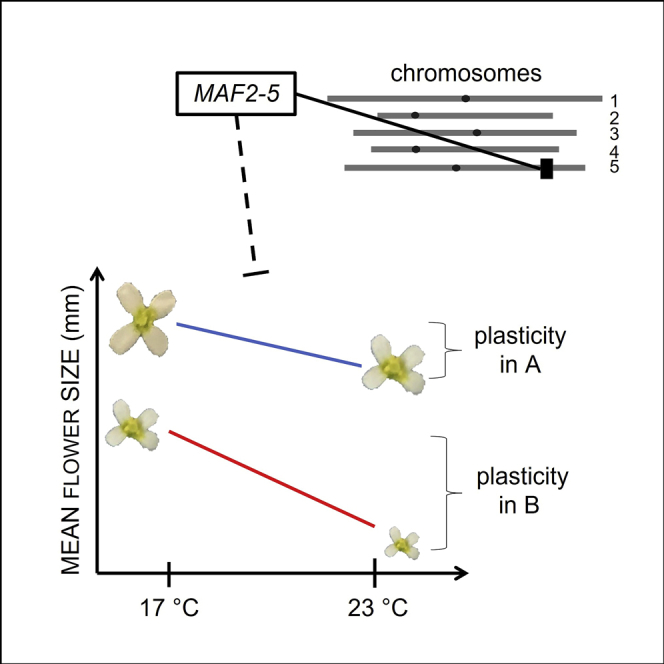

## Introduction

Global temperatures are estimated to increase by up to 5°C by the end of this century (https://climate.nasa.gov), and plants as sessile organisms must adapt to this imminent change to ensure survival (Sommer, 2020). Adaptation through natural selection relies on accumulation of mutations, which is usually a slow process. A faster way to adapt to environmental change is via phenotypic plasticity, which denotes the ability of a genotype to exhibit different phenotypes in different environments (Bradshaw, 1984; Laitinen and Nikoloski, 2019; Pigliucci, 2005). Plasticity of a focal trait in response to specific environments is in itself a quantitative trait (Duarte et al., 2021; Ordas et al., 2008; Ronnegard and Valdar, 2012; Shen et al., 2012).

In plants, traits related to growth and development of different plant organs are known to exhibit plasticity to temperature (Casal and Balasubramanian, 2019). A well-studied example in various plant species is the plasticity in flowering time that prevents plants to flower when is still too cold to survive (Barnes et al., 2022; Cao et al., 2021; Guo et al., 2018). Temperature has also been shown to affect traits directly related to reproduction such as flower size and petal number (Brondum and Heins, 1993; Hoover et al., 2012; Kang and van Iersel, 2001; Mahmood et al., 2000; McKim et al., 2017; Moccaldi and Runkle, 2007; Niu et al., 2001). As a result, these flower-related traits have been thought as highly stable (Lempe et al., 2013; Stearns and Kawecki, 1994). Yet, the natural variation and genetic basis for temperature-mediated flower size plasticity has so far not been systematically studied.

Plasticity in flower size is directly related to the reproductive strategy of the plant and change in flower size would have a direct influence on plant fitness. Outcrossing species need to attract pollinators and often have larger and colorful flowers in comparison to selfing species (Davis et al., 2008; Endress, 2011). In outcrossers, any change in flower size could have dramatic effect on the pollination, while in selfing species the size of the flower might not be as important. In fact, one might hypothesize that in predominantly selfing plants, such as *A*. *thaliana*, that are still capable of outcrossing, larger flowers could be beneficial in changing environments to increase outcrossing and thereby genetic heterozygosity.

Flower size is regulated by the floral meristem size, conversion in flower organ identity, and flower organ growth (Irish, 2010; Krizek and Anderson, 2013; Moyroud and Glover, 2017). Mutants with altered meristem function typically result in altered flower organ number. For example, *wuschel* mutants fail to maintain meristems and have a reduced number of flower organ primordia (Laux et al., 1996), while *clavata*, *wiggum*, and *ultrapetala* mutants display increased meristem size and have more flower organs (Clark et al., 1997; Fletcher, 2001; Running et al., 1998). Petal growth and petal final size are determined by differential activities of cell proliferation and division (Czesnick and Lenhard, 2015; Krizek and Anderson, 2013). Cell elongation in the basal part of the petal mainly determines the final size of this organ and many of the genes involved in regulating the final size of the petal are known (Irish, 2010; Krizek and Anderson, 2013). Whether flower size plasticity is controlled by independent genetic mechanism than flower size itself is not yet known.

Here, we first asked if flower size plasticity to temperature is found in both selfing and in outcrossing species nature. To this end, we grew and compared the temperature-mediated change in flower size among individuals of selfing *A*. *thaliana* and outcrossing *A*. *arenosa* species collected from same location. We found a higher degree of plasticity and larger amount of phenotypic variance explained by the genotype-by-environment interaction in *A*. *thaliana* than in *A*. *arenosa*. We then asked if the flower size plasticity is controlled by genes known to control flower size or by an independent genetic mechanism. Our genome-wide association analysis revealed that *MADS AFFECTING FLOWERING* (*MAF) 2–5* locus was associated to temperature-mediated flower size plasticity and our mutant analysis further confirmed the role of *MAF2-5* gene cluster in flower size plasticity to temperature. We further showed that the change in meristem size, cell number, cell size, and metabolic phenotype of flowers did not explain the flower size plasticity to temperature suggesting an independent mechanism controlling flower size plasticity and the trait itself. Together, the collection of results provides unique insights on genetic and molecular factors affecting a trait that are important for understanding adaptive responses to future environments.

## Results

### Flower size plasticity is more pronounced in selfing *A*. *thaliana* than in outcrossing *A*. *arenosa*

In outcrossing plants, large flowers are essential to attract pollinators. In contrast, flowers of selfing plants flower are often smaller. Consequently, change in flower size due to environmental perturbation could have stronger negative effects in outcrossing species. On the other hand, in selfing species, change in flower size might increase outcrossing and heterogeneity and therefore may be beneficial. Based on this, we first asked if flower size plasticity to temperature exists in natural populations and then hypothesized that it depends on the reproductive strategy of the plant. To this end, we collected seeds from the 12 individuals of the selfing *A*. *thaliana* and the outcrossing *A*. *arenosa* populations that were found to co-habit in the city of Berlin, Germany. We grew three individuals from each of the 24 mother plants at 17°C and 23°C (Table S1). We then measured flower diameter, as the average of two diagonals, from at least six open flowers from two individuals. Flower diameter was found to correlate with the width and the length of the petals and therefore it was used a proxy for flower size (STAR Methods, Figure S1). In addition, we quantified the flower size plasticity in each population by the percentage of change in flower diameter at 23°C in comparison to the flower diameter at 17°C (STAR Methods, Table S1).

**Figure 1:**
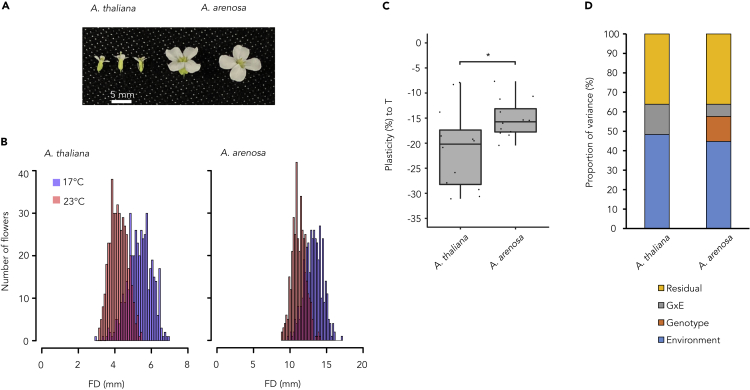
Natural populations of selfing and outcrossing Arabidopsis species (A) *A. thaliana* and *A. arenosa* flowers from natural populations grown under controlled conditions. Scale bar represents 5 mm. (B) Distribution of flower diameter at 17°C and 23°C for *A*. *thaliana* and *A*. *arenosa* flowers. (C) Box plot of the percentage of change between 23°C and 17°C. Statistical difference between means (*t*-test, p-value < 0.05) is represented with ∗. (D) Proportion of variance contributed for flower diameter in selfing *A*. *thaliana* and outcrossing *A*. *arenosa* by genotype (G), environment (E), G x E, and residual error.

Typical for outcrossers, *A*. *arenosa* flowers were considerably larger than those of *A*. *thaliana* (Figures 1A and 1B). Both *A*. *arenosa* and *A*. *thaliana* showed significant shifts in the mean flower diameters between 17°C and 23°C (*t*-test, p-value < 0.01, Figure 1B) indicating flower size plasticity to temperature in both populations. Furthermore, the larger average flower size decrease (21.1. %) at 23°C relative to 17°C in *A*. *thaliana* in comparison to the *A*. *arenosa* (15.2%) with significantly different distributions of flower size plasticity to temperature (Wilcoxon rank-sum test, p-value = 0.012, Figure 1C) points to a more pronounced temperature-mediated flower size plasticity in selfing *A*. *thaliana* than in outcrossing *A*. *arenosa*.

We further asked whether the observed flower size plasticity had genetic basis. We analyzed the proportion of variance among the *A*. *thaliana* and *A*. *arenosa* populations that was due to genotype (G), environment (E), and genotype-by-environment (G x E) effects (Figure 1D). In both species, the E effect was comparable, with 48.3% in *A*. *thaliana* and 44.7% in *A*. *arenosa*. As expected, reflecting to the genetic similarity of the selfing *A*. *thaliana* individuals at one collection site, we did not find any G component for flower size. In contrast, genotype effect explained 12.9% of variance in flower size in outcrossing *A*. *arenosa*. Furthermore, in comparison to *A*. *arenosa*, we found larger G x E component (15.5 %) for the variance in flower size in *A*. *thaliana*, which matched the summed variance due to G and G x E in *A*. *arenosa*. These results suggest that there was a genetic factor controlling temperature-mediated flower size plasticity in both species, but it was more pronounced in *A*. *thaliana* than *A*. *arenosa*.

### Genetic basis of flower size plasticity to temperature

To further identify the genes underlying temperature-mediated flower size plasticity, we utilized a genome-wide association (GWA) approach on a panel of 290 *A*. *thaliana* accessions (Table S2). To improve the statistical power of the subsequent GWA analysis, we included both accessions from different geographic locations as well as accessions with the same origin (Table S2). Accessions were grown at 17°C and at 23°C, both naturally occurring temperatures for *A*. *thaliana* (Fournier-Level et al., 2011; Hancock et al., 2011; Lasky et al., 2012), and flower size and its plasticity were measured as described above. We also asked if flower size plasticity was linked to the overall plasticity of the plant, and therefore for each accession and temperature treatment, in addition to the flower diameter, we measured two other traits, namely rosette diameter and flowering time (FT) (Figures S1 and 2A, STAR Methods).

**Figure 2:**
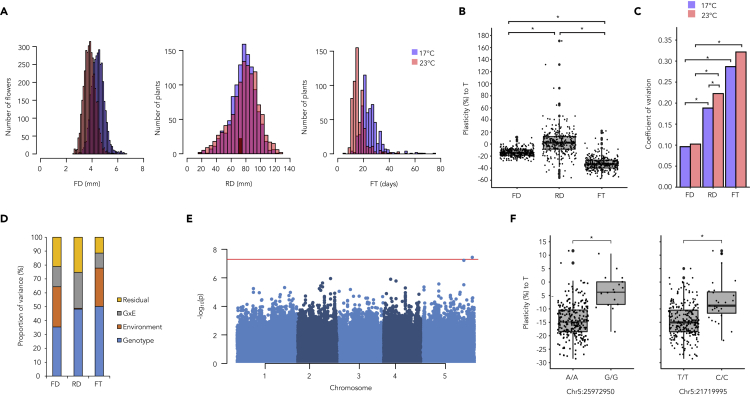
Genetic basis of flower size plasticity in *A. thaliana*. (A) Distribution of flower diameter (FD), flowering time (FT), and rosette diameter (RD) at 17°C and 23°C for 290 Arabidopsis accessions. T-test with p-value <0.05 was used to test the difference in means of FD, FT, and RD at 17°C and 23°C. (B) Trait plasticity as determined by percentage of change in a trait value between 23°C and 17°C for the same accessions. Wilcoxon rank-sum test with p-value < 0.01 was used to test significance of the distributions. (C) Coefficient of variation (CV) for the traits under differing temperature conditions. Significance was tested using bootstrap test (Amiri and Zwanzig, 2011) with p-value < 0.01 and B = 10,000). (D) Proportion of variance contributed for each flower diameter (FD), flowering time (FT), and rosette diameter (RD) trait by genotype (G), environment (E), G x E, and residual error for 290 Arabidopsis accessions grown at varying temperature. (E) Manhattan plot of significant SNPs in genomic wide association analysis for plasticity of FD. (F) Distribution of the flower size (FD) plasticities (percentage of difference in FD between 23°C and 17°C) to temperature change for the major and minor alleles for the two most significant SNPs. (t-test with ∗p < 0.05).

We found that the flower size plasticity between 23°C and 17°C varied from −28.5% to 11.6% (Figure 2B). The distributions of flower size plasticity to temperature were significantly different from those of rosette diameter or FT (Wilcoxon rank-sum test, p-value < 0.001; Figure 2B). In addition, 71.4% of the accessions showed significantly different flower size between 17°C and 23°C (*t*-test, p-value < 0.01). The majority (70.6%) of accessions with significant changes had smaller flowers at 23°C than at 17°C (Figures 2A and 2B). Despite vernalization, which synchronizes the FT, the majority (97.9%) of accessions flowered faster at higher temperature, reflecting to the known effect of temperature on this trait (Figures 2A and 2B). However, when we calculated the coefficient of variation (CV) over the 290 accessions for each trait in each temperature separately, the largest CVs were found for FT, while flower size was significantly more stable than rosette diameter or FT at the respective temperatures (p-value < 0.01, Bootstrap test (B = 10,000); Figure 2C). Therefore, we concluded that while flower size is relatively stable among accessions grown at a given temperature, they show a substantial amount of temperature-mediated flower size plasticity.

To investigate the genetic basis of flower size, rosette diameter and FT plasticities, we first partitioned the variance of these traits into its components. We found that the three investigated plasticities exhibited a degree of variance due to genotype-by-environment (G x E) interaction, namely, 14.7% for flower size, 10.9% for FT, and 25.9% for rosette diameter (Figure 2D). Interestingly, the non-residual variance in rosette diameter was largely attributed to G and G x E. These findings point at a heritable component for plasticity to temperature in all three focal traits, supported by the G x E variance component. To reveal the genetic basis for flower size plasticity, we performed GWA analysis using the percentage of change in flower diameter between 23°C and 17°C as a modeled response (see STAR Methods). We found that two SNPs were significantly associated with the flower size plasticity to temperature (Bonferroni multiple testing correction at α = 0.05, Figure 2E). One SNP was located on chromosome 5 at the position 21.72 Mb and the other at position 25.97 Mb. For both loci, the reduced flower size plasticity was associated with the minor alleles (Figures 2E and 2F). Furthermore, considering the 45-kb genomic regions in the vicinity of the two associated SNPs, we identified nineteen candidate genes controlling flower size plasticity to temperature (Table S3). To investigate the inheritance of the flower size plasticity to temperature, we performed reciprocal crosses of accessions showing: (i) the largest decrease (Tü-KS-7), (ii) an intermediate decrease (Col-0), and (iii) the lowest decrease (Bozen-1.2) in flower size at 23°C in comparison to 17°C. Analysis of these crosses demonstrated that the flower size plasticity to temperature is dominantly inherited trait (Figure S2).

### The *MAF2-5* locus is associated with flower size plasticity to temperature

**Figure 3:**
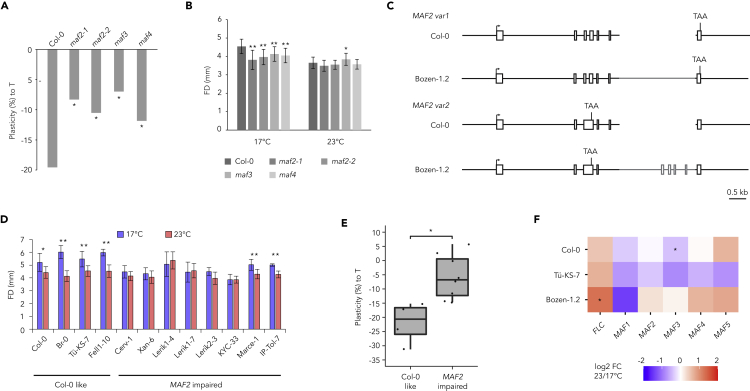
The role of *MAF2-5* locus in flower size plasticity (A) Plasticity (% of difference between 23°C and 17°C) in flower size to temperature in the *maf* mutants and wild type (Col-0). Significance was tested using bootstrap test (Amiri and Zwanzig, 2011) with p-value < 0.01 and B = 10,000). (B) Flower diameter for *maf* mutants and wild type (Col-0) grown at 23°C and 17°C. Error bars represent standard deviation, t-test with ∗p < 0.05, ∗∗p < 0.01 (n ≥ 12). (C) Genomic structure of the *MAF2* locus in Col-0 and Bozen-1.2 with sequenced splicing variants *MAF2 var1* and *var2* cDNAs mapped to the sequenced genomic region. Scale bar denotes 0.5 kb. (D) Flower diameter for selected accessions with either premature stop codons or absent start codons in the *MAF2* gene (n ≥ 12). Error bars represent standard deviation, t-test with ∗p < 0.05, ∗∗p < 0.01. (E) Box plots for plasticity (% of difference between 23°C and 17°C) in FD in accessions with Col-0 like *MAF2* or impaired *MAF2* gene (Wilcoxon rank-sum test with p-value < 0.05). (F) Log2 fold-change of *MAF1-5* genes in Col-0, Tu-KS-7, and Bozen-1.2 at 23°C compared to 17°C measured with quantitative RT-PCR. n = 4, p-value < 0.05 (*t*-test).

From the candidate genes, the *MADS AFFECTING FLOWERING* (*MAF*) *2–5* cluster was the only genes expressed in flowers and known to encode proteins that regulate temperature-dependent response in *A*. *thaliana* (Airoldi et al., 2015; Rosloski et al., 2013). We grew two independent T-DNA lines for *maf2* and single mutants for *maf3* and *maf4* at 17°C and 23°C. All the *maf*-mutants showed reduced plasticity in response to temperature in comparison to the WT (Figure 3A). We further identified that in all lines, the reduced plasticity was due to the significantly reduced flower size at 17°C compared to WT (Figure 3B) suggesting the role of *MAF2-5* locus in mediating flower size plasticity to temperature.

Interestingly, sequencing of the *MAF2* and *MAF3* genes and the two known splicing variants of *MAF2* locus in Bozen-1.2, which has reduced plasticity in response to temperature, revealed a ∼2 kb insertion, that was not found in the Col-0 reference genome, in the 6th intron of *MAF2*, with similarity to the *MAF3* gene (Figure 3C). In addition, Bozen-1.2 had a 422 bp insertion in the 3′ UTR of the *MAF3* gene, pointing to the different organization of the *MAF2-5* gene cluster associated with the temperature-mediated flower size plasticity. To get further insights for the role of *MAF2-5* locus in decreasing flower size plasticity, we compared the 1001 Arabidopsis genomes data sequences of the *MAF2* locus, the most polymorphic and highly expressed gene of the cluster. We identified eight accessions that either lacked the start codon or had a premature stop codon in the first exon of the *MAF2* gene. We grew these eight accessions at both 17°C and 23°C and quantified their flower size (Figure 3D). Six of these accessions did not significantly change their flower size when grown at 23°C or at 17°C (*t*-test, p-value < 0.05, Figure 2D). The distribution of the flower size plasticities to temperature in accessions with Col-0-like *MAF2* was significantly different from the distribution in accessions with impaired *MAF2* (Wilcoxon rank-sum test, p-value < 0.01, Figure 3E). While the accessions in each group differ in other parts of the genome, this result further supports the role of *MAF2-5* locus responsible for flower size plasticity to temperature that was confirmed with the mutant analysis, above.

While the *MAF2-5* temperature-dependent gene expressions are well characterized in leaves, the role of temperature in their expression in flowers is not known. Therefore, we investigated if the *MAF2-5* genes were differentially expressed in flowers. This analysis also included two related MADS-box genes, *MAF1*, that shows high similarity to *MAF2-5* genes and is located on chromosome 1, and *FLC*, that together with *MAF1-5* genes is known to regulate vernalization-dependent flowering (Airoldi et al., 2015; Pose et al., 2013). We measured transcript levels of the *MAF1-5* and *FLC* genes in Col-0, Tü-KS-7, and Bozen-1.2 open flowers. We did not observe significant changes in the expression levels correlating with the degree of plasticity between the two temperatures (Figure 3F) indicating that the flower size plasticity was not explained by the different amounts of *MAF2-5* transcripts.

### Flower size plasticity is uncoupled from rosette size plasticity

**Figure 4:**
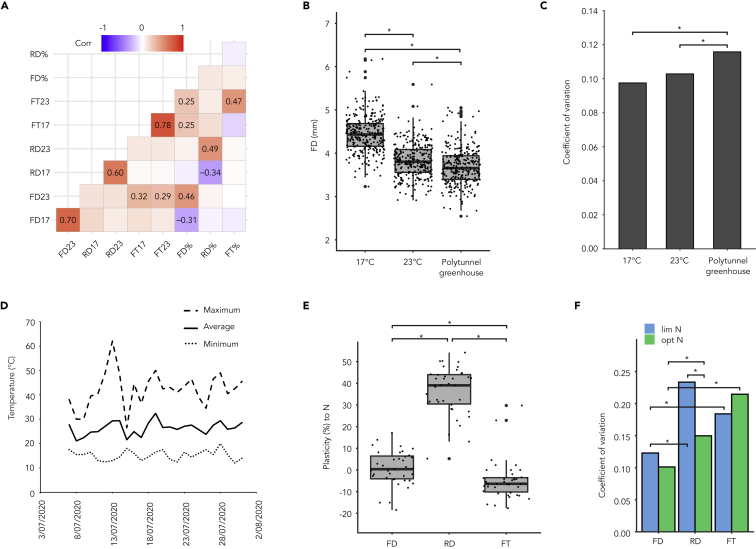
Flower and rosette size plasticities to temperature are uncoupled (A) Pearson correlation with Bonferroni correction of traits for the 290 accessions at 23°C and 17°C; corrected p-value < 0.05. (B) Box plot of flower diameter in plants grown either 17°C, 23°C or at polytunnel greenhouse. (p-value < 0.01, Wilcoxon rank-sum test). (C) Coefficient of variation (CV) of flower diameter across the accessions grown in each of the conditions. (D) A graph showing the minimum, maximum, and average temperature during the experiment. (E) Trait plasticity comparing optimal and limited nitrogen for 39 *A. thaliana* accessions covering the range of flower size plasticities to temperature change. (p-value < 0.01, Wilcoxon rank-sum test). (F) Coefficient of variation for the traits under differing nitrogen conditions. In C and F, the significance was tested using a bootstrap method (Amiri and Zwanzig, 2011 with p-value < 0.01 and B = 10,000).

Motivated by these findings, we asked if flower size plasticity reflects the overall plasticity of the plant, measured in different traits, or whether plasticity in response to temperature of different traits is independently regulated. To test this, we investigated if there is any significant association between the mean values of the flower size, rosette diameter, and FT as well as their plasticity to temperature. First, we determined the Pearson correlation coefficient between these traits over the accessions (Figure 4A). The significant correlations between flower size and rosette diameter at the two temperatures with their respective plasticities indicated that accessions with larger flowers and rosettes at 17°C were more stable when switched to 23°C (Figure 4A). While no correlations between flower size and rosette diameter were observed, FT at 17°C and 23°C correlated significantly (p-value < 0.05 with Bonferroni correction) with both flower size at 23°C and flower size plasticity (Figure 4A). Moreover, the lack of correlation between plasticities of the different traits suggests that they may be independently regulated.

Second, we asked, if even higher and naturally fluctuating temperature may further increase the studied plasticities. We measured flower size in the full set of 290 accessions in a polytunnel greenhouse, in which the average temperature was 26.3°C, with strong fluctuations ranging between 12°C and 62°C (Figures 4B and 4D). Comparison of the distributions of flower sizes over the accessions in each of the three scenarios showed that their means were statistically different between 17°C and polytunnel as well as between 23°C and polytunnel (p-value = 0.0001) (Figure 4B). However, the accessions grown in the polytunnel exhibited larger CV in comparison to the growth under the two constant temperatures (p-value = 0.0423, Bootstrap test, B = 10,000, Figure 4C). These results indicated that increased natural temperature fluctuations further decreased the flower size and flower size plasticity but also increased variability among the accessions.

### Flower size plasticity occurs in response to specific environments

We then hypothesized that plasticity in flower size and rosette diameter is also uncoupled under environmental conditions that are known to promote plasticity in rosette diameter. To test this, we grew a subset of 39 accessions covering the range of flower size plasticity to temperature at two nitrogen (N) conditions, one optimal and one limiting for rosette diameter (Duarte et al., 2021; Pandey et al., 2019). We quantified the plasticity to N as the percentage of change in flower size, rosette diameter, and FT at optimal N in comparison to the traits at limiting conditions for each accession (Table S4). Our data demonstrated that the distribution of flower size plasticity to N availability was significantly different from the distributions of rosette diameter and FT plasticity to N availability (p-value < 0.01, Wilcoxon rank-sum test, Figure 4E). While 33.3% of the accessions exhibited significantly larger rosette diameter when grown at optimal N in comparison to limiting N, 12.8% of the accessions had significantly larger and 7.7% had smaller flower size when grown at optimal N compared to limiting N (*t*-test, p-value < 0.05). None of the accessions showed significant change in FT with the changing N conditions. Furthermore, the flower size plasticity to temperature and to N availability was not significantly correlated (r = 0.224, p-value = 0.17), indicating that they are controlled by different genetic and molecular networks. Like the findings from the temperature experiments, the CVs of flower sizes at the two N conditions were significantly smaller than for those of FT and rosette diameter at the respective conditions (p-value < 0.05; Bootstrap test, B = 10,000, Figure 4F). Taken together, we concluded that the plasticities in flower size and rosette diameter are uncoupled from each other and the degree of plasticity strongly depends on the environmental conditions.

### Role of meristem size and petal growth plasticity in flower size plasticity to temperature

**Figure 5:**
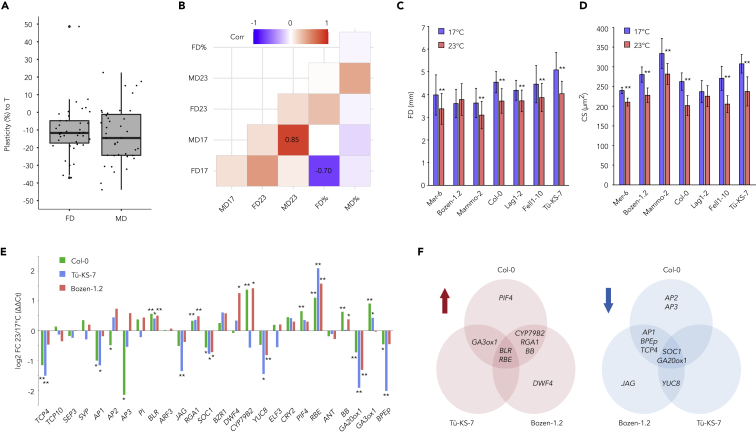
Transcriptional and cellular mechanisms associated with flower size plasticity (A) Box plots showing the percentage of difference between 23°C and 17°C in meristem diameter (MD) and flower diameter (FD). (B) Pearson correlation of meristem and flower diameters in plants grown at 23°C and 17°C and their plasticities. p-value < 0.05. (C and D) FD and D) cell size (CS) in seven *A. thaliana* accessions grown at 23°C and 17°C. T-test with p-value < 0.01 was used for significance (∗∗). (E) Transcript profiling of open flowers using quantitative RT-PCR of 26 genes with known involvement in temperature dependent growth or development, in Col-0, Tü-KS-7, and Bozen-1.2. To test significance, t-test with p-value < 0.01 (∗∗) and < 0.01 (∗) between 23°C and 17°C in each accession was used. (F) Venn diagram comparing the transcript showing significantly more expression (*t*-test, p-value < 0.05) in Col-0, Tü-KS-7, or Bozen-1.2 flowers of plants grown at 23°C than in flowers grown at 17°C.

Flower size is regulated by the floral meristem size, conversion in flower organ identity, and by organ growth, due to modifications in the duration or rate of the cell expansion and cell division (Czesnick and Lenhard, 2015; Huang and Irish, 2016). To examine whether flower size plasticity to temperature depends on the plasticity of the meristem size or cell size measured as the epidermal cell surface area, we selected a subset of 39 accessions covering the range of flower size plasticities to temperature (Table S5). Like flower size, we observed that most accessions (76.9%) had reduced meristem size, measured with the meristem diameter, at 23°C in comparison to 17°C, with only 12.8% exhibiting significant decrease (Figure 5A). Yet, there was no correlation between the absolute size and the degree of decrease between flower size and meristem size (corrected p value < 0.05; Figure 5B). Next, to investigate the role of cell elongation and division in flower size plasticity to temperature, we measured the cell number and size in petals of seven selected accessions and found that independent of their flower size plasticity (Figures 5C and 5D, and S3), cells were smaller at higher temperature in all accessions except Lag1-2 (Figures 5C and 5D). These results showed that the cell size did not explain the growth plasticity in petals, but it is rather due to the rate or duration of cell division.

To further investigate the mechanisms regulating the flower size plasticity to temperature, we evaluated whether the expression of 26 genes known to regulate petal growth depends on temperature (Table S6). We reasoned that although this analysis would not capture the post- transcriptional regulation of the genes involved in petal growth, it could give indications of the transcriptional networks associated with the temperature-mediated growth in petals. We pooled flowers sampled from the accessions showing the largest decrease (Tü-KS-7), the smallest decrease (Bozen-1.2), and the intermediate decrease (Col-0) in flower size at 23°C in comparison to 17°C. To identify genes showing accession-specific response patterns, we identified and compared statistically significant differentially expressed genes (*t*-test, p-value < 0.05) between 23°C and 17°C in each accession (Figures 5E and 5F). The expression analysis showed that in both accessions with a decrease in flower size (i.e., Col-0 and Tü-KS-7), *BPEp*, *TCP4*, and *AP1* were significantly downregulated at 23°C in comparison to 17°C (Figures 5E and 5F), while *GA3ox1* was significantly upregulated. These results suggest that the decreased growth at 23°C is associated with auxin and gibberellic acid transcriptional networks that regulate petal identity and growth (Gregis et al., 2009; Nag et al., 2009; Szecsi et al., 2006). In Bozen-1.2, the only gene with altered (upregulated) expression was *DWF4*, indicating that the altered flower size plasticity could be associated with brassinosteroid signaling (Choe et al., 1998), implicated in regulation of plant growth (Singh and Savaldi-Goldstein, 2015).

### Plasticity in flower primary metabolism to temperature

Significant changes in the floral metabolome have previously been measured in Col-0 flower buds and mature flowers exposed to high temperature (Borghi et al., 2019). This prompted us to investigate plasticity of primary metabolism in response to temperature change in flowers. We grew 11 accessions found in our earlier experiments to increase, decrease, and to maintain relatively constant flower size at 23°C in comparison to 17°C and measured levels of primary metabolites (Table S7 and Figure S4). Principal component analysis (PCA) based on the metabolite levels showed separation of the accessions in response to temperature across PC2, explaining 22.3% of the variance (Figure S5A). Furthermore, PC1, explaining 32.2% of the variance, negatively correlated with the latitude of the collection of the accession (r = −0.55, p-value = 0.008), but not longitude (r = 0.017, p-value = 0.94) of origin. In addition, PCA of the log2 fold-change of metabolites indicated that accessions with increasing and not changing flower size plasticity to temperature were separated from the decreasing (except for Eden-2) based on PC2, explaining 21.7% of the variance (Figure S5B).

Next, we used ridge regression to model flower size plasticity to temperature based on the plasticity in the levels of 55 measured metabolites, resulting in a cross-validated mean squared error for the minimum lambda of 0.0112. Based on the magnitude of the coefficients (in absolute value), we found that plasticity contributed most to the variance explained in key sugar phosphates, e.g. fructose-6-phosphate and glucose-6-phosphate, carboxylic sugar (i.e. inositol), and other primary metabolites (e.g. glycerol, glycerol-3-phosphate (an essential component of glycerolipids), and glycolate (found to associate with bulb size in *Lilium longiforum* (Lazare et al., 2019)) (Table S8). In addition, plasticities in phenylalanine and proline, as proteinogenic amino acids, were found to contribute to the variance explained. These results revealed that indeed the floral metabolism, and especially sugars, shows plasticity in response to temperature. Yet, further experiments are required to understand whether and how the metabolism is responsible for the observed plasticity in petal growth.

## Discussion

The large degree of variation in flower size, shape, and color between different species allows niche separation and successful pollination, while the stability of floral traits within one species assures successful pollination and reproduction (Davis, 2008; Davis et al., 2008; Irish, 2010; Krizek and Anderson, 2013; Moyroud and Glover, 2017). Consequently, any change in the flower structure due to climate change could affect the adaptability of plant populations. We showed that there is flower size plasticity in response to warmer temperature in individuals of co-occurring populations of selfing *A*. *thaliana* and outcrossing *A*. *arenosa* species. Yet, the flower size plasticity was more pronounced and had stronger genetic basis in selfing *A*. *thaliana*, which was used for further studies. By using the genetic resources available for *A*. *thaliana*, we further identified that *MADS AFFECTING FLOWERING 2–5* (*MAF2-5)* gene cluster was responsible for temperature-mediated flower size plasticity. Moreover, we found that plasticity was not explained solely by changes in meristem or petal epidermal cell surface area and number indicating another cellular mechanism of temperature-mediated growth in flowers and plasticity of their size. The independent mechanism controlling plasticity and the focal trait itself was further supported by the lack of overlapping association in the GWA analysis performed for temperature-mediated flower size plasticity and to flower size at a given temperature (Figure S3).

*MAF2-5* genes are known to encode proteins that regulate temperature-dependent flowering time in *A*. *thaliana*. In leaves, the *MAF2-5* genes have overlapping roles in regulating flowering time in response to vernalization (Gu et al., 2013) and they can function in a compensatory manner by altered expression relative to one another during the vernalization or when overexpressed (Ratcliffe et al., 2003). We found that *maf2-4* mutants showed reduced plasticity in comparison to Col-0 WT indicating that *MAF2-5* gene cluster could act redundantly in control of flower size in a similar manner to how the *MAF2-5* cluster regulates flowering time in response to vernalization (Airoldi et al., 2015; Rosloski et al., 2013).

The alternative splicing of the *MAF2-5* cluster has been proposed to facilitate rapid adaptation to changes in ambient temperature in plants (Theissen et al., 2018). In leaves, temperature-dependent alternative splicing of *MAF2* has been shown to mediate the flowering response (Airoldi et al., 2015). For *MAF2*, at low temperatures in leaves, variant 1 (var1) is the most abundant splice variant that by interacting with SHORT VEGETATATIVE PHASE (SVP) represses flowering; in contrast, at high temperatures in leaves, variant 2 (var2), which cannot interact with SVP, is the most abundant, and flowering is induced (Airoldi et al., 2015; Rosloski et al., 2013). It has been further hypothesized that in a similar way as the control of flowering time by the different variants of another member of *MAF* gene family, namely FLM, also MAF2-5 cluster could mediate plasticity in flowering time without negative pleiotropic effects (Lutz et al., 2017; Theissen et al., 2018). Our study showed that the *MAF2-5* cluster is also involved in controlling plasticity in flower size to temperature. It is possible that alternative splicing of the *MAF2-5* genes also mediates the temperature-dependent plasticity in flowers. In future, complementation experiments with the different *MAF2-5* alleles in different background are required to be able to fully conclude the role of *MAF2-5* gene cluster in temperature-mediated flower size plasticity.

Our evidence points at independent genetic and molecular mechanisms controlling flower size plasticity and flower size itself and supports the contribution of phenotypic plasticity to evolutionary events such as adaptation and diversification (Bradshaw, 1984; Laitinen and Nikoloski, 2019; Pigliucci, 2005; Sommer, 2020; Valladares et al., 2006). We showed that in nature, *MAF2-5* alleles resulting in increased plasticity were dominant and more common, while the Bozen1.2-like alleles of *MAF2-5* gene cluster associated with reduced plasticity at the lower temperature were rare. On one hand, plasticity can increase the success of an individual in adapting to spatial and temporal variability of environments; on the other hand, it could decrease the adaptive genetic variation of populations by masking the genetic differences from selection. Yet, it remains to be investigated whether flower size plasticity is due to selection pressure acting on this trait directly or is a result of pleiotropy. Our results discovered that temperature-mediated flower size plasticity is genetically controlled, that flower size plasticity was more common than stability among global *A*. *thaliana* accessions, and that the increased plasticity was dominantly inherited in these accessions. These point toward an adaptive value of temperature-mediated flower size plasticity in *A*. *thaliana*.

To conclude, our findings challenge the current thinking of flower size as a highly robust trait and highlight the importance to understand and study the impact of climate change on floral traits and plant reproductive strategies.

### Limitations of the study

We conducted a first systematic study of flower size plasticity in response to an increase in ambient temperature. We show that a known flowering time regulator, *MAF2-5* gene cluster, is also mediating flower size plasticity. Yet, the role of different *MAF2-5* alleles in regulating temperature-mediated growth in flowers was not addressed. In addition, the mode-of-function of the *MAF2-5* gene cluster in conferring plasticity was not shown in this study. Lastly, the evidence supporting the hypothesis that flower size plasticity is linked to the reproductive strategy of the plant is limited to the knowledge of co-occurring populations of outcrossing *A*. *arenosa* and *A*. *thaliana*. Further studies are required for detailed understanding of mechanism controlling flower size plasticity and its potential in steering plant adaptation.

## STAR★Methods

### Key resources table

**Table:** 

REAGENT or RESOURCE	SOURCE	IDENTIFIER
**Chemicals, peptides, and recombinant proteins**		

Phusion Taq DNA polymerase	Thermo Fisher Scientific	N/A
DreamTaq DNA polymerase	Thermo Fisher Scientific	N/A
pGEMT-Easy vector	Promega	N/A
RNeasy Plant Mini Kit	Qiagen	N/A
RevertAid First Strand cDNA Synthesis Kit	Thermo Fisher Scientific	N/A
TURBO DNase	Life Technologies	N/A
ImProm-II Reverse Transcription System	Promega	N/A

**Experimental models: Organisms/strains**		

*A*. *thaliana* accessions	This paper	Table S1
Wild *A*. *thaliana* and *A*. *arenosa* seeds	This paper	Table S1

**Oligonucleotides**		

MAF2/3 F TCCTTCGTTTTCGCATTTTGT	this study	Table S6
MAF2/3 R ATGTCGAGTTCCCTTGTGGC	this study	Table S6
MAF Full F ATGGGTAGAAAAAAAGTCGAG	this study	Table S6
MAF full R CTTGAGCAGCGGAAGAGTCTCC	this study	Table S6
Primers for qRT experiment	this study	Table S6

**Software and algorithms**		

QuantPrime	Arvidsson et al. (2008)	N/A
EasyGWAs	Grimm et al. (2017)	N/A

**Other**		

Metabolite extraction, quantification and analysis	Lisec et al. (2006); Borghi et al. (2019)	N/A
Standards for metabolite measurements	Alseekh et al. (2021)	N/A

### Resource availability

#### Lead contact

Further information and requests for resources and reagents should be directed to and will be fulfilled by the lead contact, Roosa Laitinen (Roosa.Laitinen@helsinki.fi).

#### Materials availability

This study did not generate new unique reagents. Plasmids generated in this study are available upon request.

### Experimental model and subject details

#### Plant material and growth conditions

For the population comparisons, seeds from 12 *A*. *thaliana* plants and 12 *A*. *arenosa* plants were collected from Tiergarten, Berlin in July 2020 (52.511730, 13.350308). Three progenies from each of the 24 mother plants were germinated on soil for 1 week and then vernalized for 6 weeks. Plants were then grown at LD either 23°C or 17°C. *A*. *thaliana* accessions were obtained from the Nottingham Arabidopsis Stock Center (NASC) (Table S2). In all experiments, prior sowing the seeds, they were stratified for 4 days in 0.1% agarose in the dark at 4°C. For the first screening, 290 accessions were selected and grown in 6 cm diameter pots, one plant per pot. After sowing, the pots were kept one day at 20°C/6°C under long-day (LD) conditions (16 h light : 8h dark) with a photon flux density of 250 μM/m^2^/s. Then, all pots were vernalized at 4°C for at least 5 weeks. The Swedish accessions did not flower with 5 weeks and were vernalized 8 weeks (Hovdala-2, Fäb-4, Tottarp-2, Lund, TDr-8, Sanna-2, St-0, Eden-2, Bil-7, Bil-5, Eden-9, Eden-1, Eden-7, Ost-0, T620, Hov4-1, and Lis-2). After the vernalization, four plants of each accession were transplanted to individual 6 cm pots. Two plants of the same accession were grown at 17°C and two at 23°C under LD conditions with photon flux 140 μM/m^2^/s and relative humidity 70%. The pots were randomized on trays and the trays were rotated every second day. In the greenhouse experiment, the pots were vernalized and grown in polytunnel greenhouse in June 2019 without humidity or temperature control.

To investigate the flower size plasticity in response to N availability in soil, the soil was complemented with two different amounts of nitrogen, optimal and limited, as described in Pandey et al., (2019) and Duarte et al., (2021). The seedlings from 39 accessions, selected to cover the different flower size plasticities (Table S4), were vernalized for 7 weeks and grown in four replicates in both soil conditions at LD and 23°C. T-DNA lines, SALK_045623 (*maf2-1*), SALK_141778 (*maf2-2*), SALK_044822 (*maf3*) and SALK_028506 (*maf4*), were ordered from NASC, and isolated to homozygosity using primers designed with SALK T-DNA express (http://signal.salk.edu/tdnaprimers.2.html; Table S6).

### Method details

#### Trait measurements

290 accessions were measured for flower diameter (FD), rosette size (RD), and flowering time (FT) (Table S1). For FD, at least six open flowers from two individuals per accession were collected from the primary inflorescence. To minimize the technical variation, flowers were taken after the 8^th^ flower had opened and were always harvested in the morning after the lights had been on for at least three hours. Flowers were placed on a 96-well plate containing 1–2% agarose. Flower diameter was measured from pictures as an average of two diagonal measurements. We confirmed that flower diameter was strongly positively correlated with flower area (p-value < 0.05, with values ranging from r = 0.88 in both 17°C and in 23°C) and petal width (with values ranging from r = 0.60 in 17°C to 0.69 in 23°C), and we used flower diameter as a proxy for flower size (Figure S1). Rosette diameter (as an average of two diagonal measurements) was measured from pictures taken above at the time of flowering. Both flower diameter and rosette diameters were measured using ImageJ. FT was the number of days from the transferring of the plants to the growth chamber until the day of bolting. The percentage of change in each trait was calculated as the difference between the mean values 23°C vs. 17°C, divided by the mean value at 17°C and multiplied by 100. The coefficient of variation among accessions in the two temperature conditions were measured as the standard deviation of the mean values of all accession divided by the average of the mean values of all accessions.

#### Flower area, petal width and meristem and cell size measurements

A subset of 39 accessions were selected to cover the range of different flower size plasticities to temperature and were grown for more detailed phenotyping (Table S5). For the meristem size measurements, one meristem of three plants plant was carefully exposed under stereo microscope using sharp forceps from a flower bud of the main inflorescence when the stem was 2–3 cm (Leica MZ12), and digital microscope (Keyence VHX-7000) was used for imaging. The diameter of the main meristem was used as a measure for the meristem size. Simultaneously the accessions were grown in the same chamber, and at least 14 flowers from each accession in each temperature were measured for flower diameter, flower area and petal width. Cell size, measured as epidermal petal epidermal cell surface area and cell number were measured from microscope images of flowers of three plants of every accession. For each plant, number of cells were counted cells in the base and the tip of the petal in area of 0.1 cm^2^. For the cell size, the area was divided by the number of cells and the average of the 12 measurements were used.

#### Genome wide association (GWA) analysis

From the 290 accessions that we used for the phenotypic analysis; 280 accessions were fully sequenced by the 1001 Genomes Project. The statistical power of the GWA analysis was improved by including both the accessions originating world-wide and sets of those originating from a single place. GWA analysis of flower size plasticity was conducted using the easyGWAS (Grimm et al., 2017) (https://easygwas.ethz.ch/) and percentage of change at 23°C in comparison to 17°C of the FD was used as the trait. The full sequence of the accessions (1001genomes.org/) and with TAIR10 as gene annotation set, all available SNPs for *A*. *thaliana* were selected with a minor allele frequency (MAF) > 5%. Association analysis were performed with EMMAX (Kang et al., 2010). Zero mean transformation for the percentage of change at 23°C in comparison to 17°C of the flower size and log10 transformation of mean FD at 17°C and 23°C was applied. A Bonferroni correction with a nominal significance threshold (α) of 0.05 was applied, corresponding to a p-value of 2.61 × 10^−7^. Manhattan plots were drawn with the qqman package in R.

#### Sequencing of the *MAF2-MAF3* locus

Genomic DNA was extracted from leaf tissue with the CTAB method (Clarke, 2009). The *MAF2-MAF3* locus was amplified from Col-0 and Bozen-1.2 DNA with forward primer, 5′- TCCTTCGTTTTCGCATTTTGT-3′ and reverse primer, 5′-ATGTCGAGTTCCCTTGTGGC-3′ using proof reading Phusion Taq DNA polymerase and then A-tailed with DreamTaq DNA polymerase (Thermo Fisher Scientific). PCR fragments were ligated into pGEMT-Easy vector (Promega) and isolated. Primers used for Sanger sequencing of the *MAF2-MAF3* locus are listed in Table S6. For cloning of *MAF2* cDNAs, total RNA was extracted from open flowers of Bozen-1.2 and Col-0 with a RNeasy Plant Mini Kit (Qiagen), and cDNA synthesized from 1 μg of RNA with RevertAid First Strand cDNA Synthesis Kit (ThermoFisher Scientific). Full length sequences from start to stop codon fragments were amplified with forward primer, 5′-ATGGGTAGAAAAAAAGTCGAG-3′, and reverse primer, 5′-CTTGAGCAGCGGAAGAGTCTCC-3′, then cloned and sequenced as described for the genomic fragments. Expressed cDNA sequences were mapped to the derived genomic sequences for Col-0 and Bozen-1.2 to determine intron-exon structures.

#### Expression analysis

For the expression analysis, total RNA was extracted by acid guanidinium thiocyanate-phenol-chloroform extraction method (Chomczynski and Sacchi, 2006) from pooled open flowers of three plants collected from accessions grown either at 17 or 23°C. 1 μg of the total RNA was treated with TURBO DNase (Life Technologies) and used for reverse transcription ImProm-II Reverse Transcription System (Promega). Expression was measured using qRT-PCR from flowers grown at 17 or 23°C with four biological replicates of each accession and temperature permutation, relative to *UBC*/AT5G25760 reference gene (Czechowski et al., 2005). Primers for measurement were designed with QuantPrime (Arvidsson et al., 2008) and are listed in Table S6. Transcripts were deemed to be differential expressed if they were statistically significantly different by t-test, p < 0.05.

#### Metabolic analysis

Primary metabolites were extracted from 11 accessions for showing increased, decreased, or constant flower diameter in response to temperature. For each accession, 4 replicates each constituted of a pool of open flowers collected (appr. 10mg) from three plants were used for the analysis. Metabolite extraction, derivatization and profiling were carried out exactly as described previously (Lisec et al., 2006). Metabolites were analyzed as described in (Borghi et al., 2019) and data was reported following current reporting standards (Alseekh et al., 2021).

### Quantification and statistical analysis

All statistical analyses were performed in R. To test the statistical significance between the difference in means and distributions, t-test and Wilcoxon rank-sum respectively were applied. For the statistical significance of the CVs and plasticity (%), a bootstrap test was applied where B = 10,000 (66). The Pearson correlations were calculated using the cor() function and p-values were Bonferroni corrected for multiple testing using the p.adjust() function. The resulting correlations were represented using the R-package ggcorplot for multiple correlations, or the ggpubr package and ggscatter() for single correlations. Using the lme4 package of R and the lmer() function, contribution of variance for each trait was calculated in a mixed linear model:

TRAIT=ENV+GEN+GEN×ENV+e

Where TRAIT was either FD, RD or FT, ENV is environmental treatment (17 or 23°C), GEN is genotype (accession), GEN∗ENV is the genotype by environment interaction, and e is the residual error. ENV was treated as a fixed effect while all other variables were assumed as random effects. For calculation of general heritability of each trait at the two temperature conditions, the data were fitted to the model with each variable a random effect:

TRAIT=GEN+e

Using the glmnet package of R and the cv.glmnet(), glmnet() and predict() functions, the contribution of the coefficients for all 55 measured metabolites towards FD17, FD23 and FD%. FD17 and FD23 models were determined in respect to log transformed metabolite intensities at the respective temperatures, while the FD% model was determined respective to plasticity of each metabolite. Minimum lambda was determined by cross validation with K = 3, and this was used as the optimal lambda value to determine metabolite factor coefficients. Mean square error for the minimum lambda is reported in the text. PCAs were calculated on log transformed metabolite intensities using the prcomp() function and represented using the factoextra package of R and the fviz_pca_ind().

## Data Availability

• All data reported in this paper will be shared by the lead contact upon request. • This paper does not report original code. • Any additional information required to reanalyze the data reported in this paper is available from the lead contact upon request.

## Supplemental information

Document S1. Figures S1–S5 and Tables S1, S3, and S8

Table S2. Flower diameter (FD), rosette diameter (RD) and flowering time (FT) data for 290 accessions in grown at 17 °C, 23 °C and polytunnel greenhouse, related to Figure 2

Table S4. Flower diameter (FD), rosette diameter (RD) and flowering time (FT) data for 39 accessions grown at optimal and limited N, related to Figure 4

Table S5. Flower diameter (FD), flower area, petal width and meristem diameter for 39 selected accessions grown at 17 °C and 23 °C, related to Figure 5

Table S6. List of primers used for qRT-PCR, genotyping and sequencing of genomic and cDNA sequences, related to Figure 5

Table S7. a. Flower diameter and b. Primary metabolites measured from 11 accessions grown at 17 °C and 23 °C, related to STAR Methods and Figures S4 and S5
