# Supplementary material for: Temperature-mediated flower size plasticity in Arabidopsis
Source: iScience. 2022 Oct 21;25(11):105411. doi: 10.1016/j.isci.2022.105411 (PMC9646949; doi:10.1016/j.isci.2022.105411)
Supplement: Document S1. Figures S1–S5 and Tables S1, S3, and S8 [file mmc1.pdf]

iScience, Volume *25*

Supplemental information

Temperature-mediated flower size plasticity in Arabidopsis

Andrew Wiszniewski, Estefanía Uberegui, Michaela Messer, Gulmairam Sultanova, Monica Borghi, Gustavo Turqueto Duarte, Rubén Vicente, Katelyn Sageman-Furnas, Alisdair R. Fernie, Zoran Nikoloski, and Roosa A.E. Laitinen

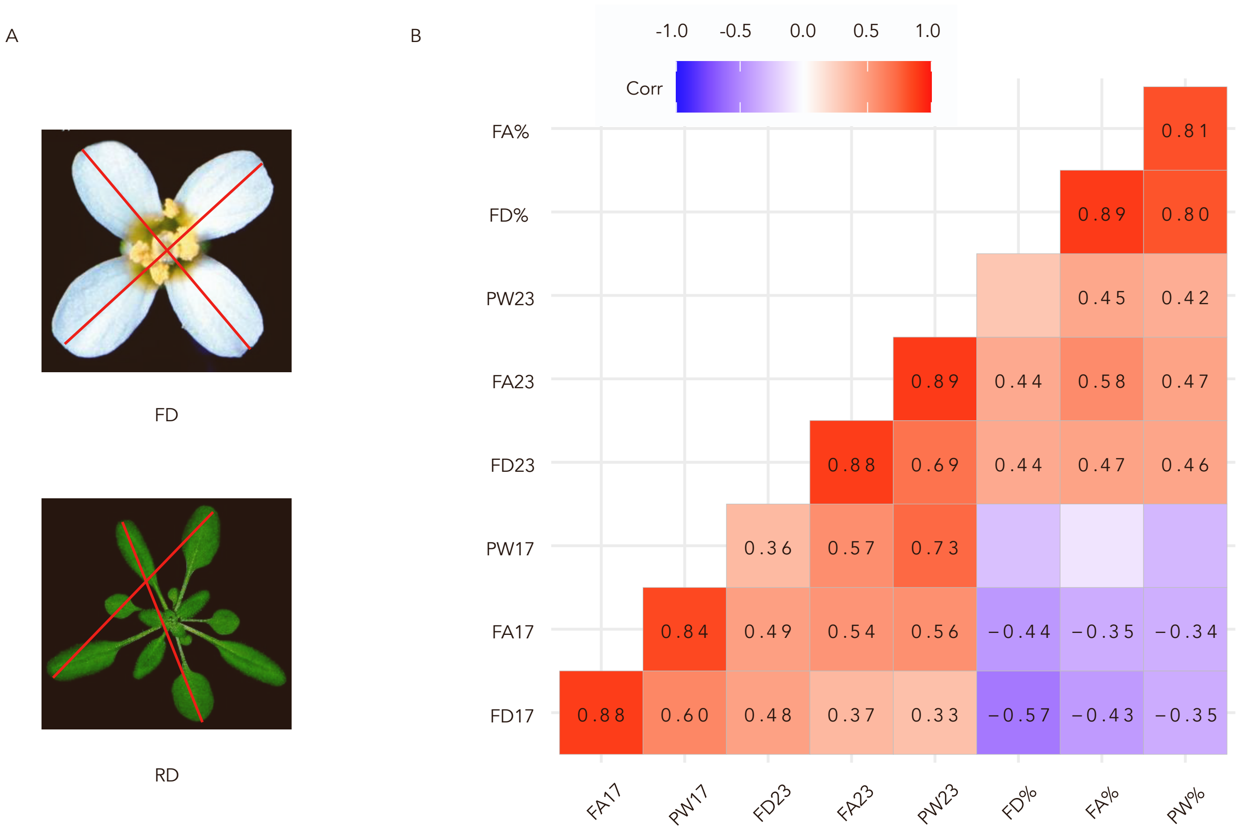

Figure S1. A) Flower diameter (FD), rosette diameter (RD) were measured using ImageJ from images taken above. For flower diameter, at least two individual plants and at least 6 flowers from each were used for measurements. For RD, two individual plants were measured. FT was measured as the days to flowering after germination. All plants were vernalized for 6-8 weeks unless otherwise stated. B) Pearson correlation of petal width, flower diameter and flower area in 39 accessions of *A. thaliana*. (p-value<0.05 where R is shown) (Table S5).

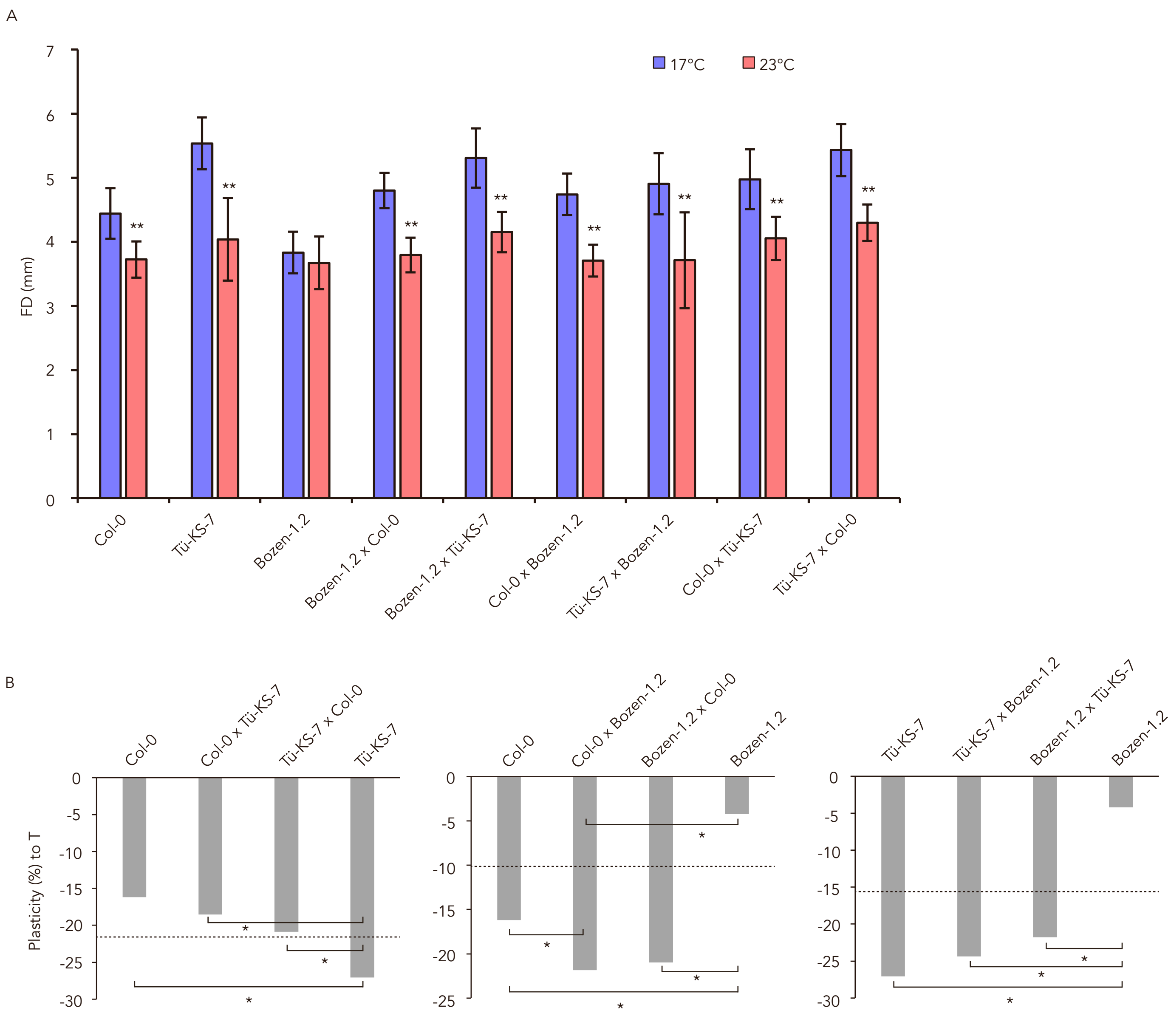

Figure S2. A) Flower diameter of parents and diallel F1 crosses among Col-0, Tü-KS-7 and Bozen-1.2 grown at 17°C and 23°C T-test with p-value < 0.05 (*), p-value<0.01 (**), p-value < 0.001 (***) was used for significance. B) Plasticity (% of difference between 23 °C and 17 °C) in flower size to temperature of parents and diallel F1 crosses (Bootstrap method,with p-value < 0.01 and B = 10,000, Amiri and Zwanzig, 2011). Dashed line represents the midpoint between the two respective parents.

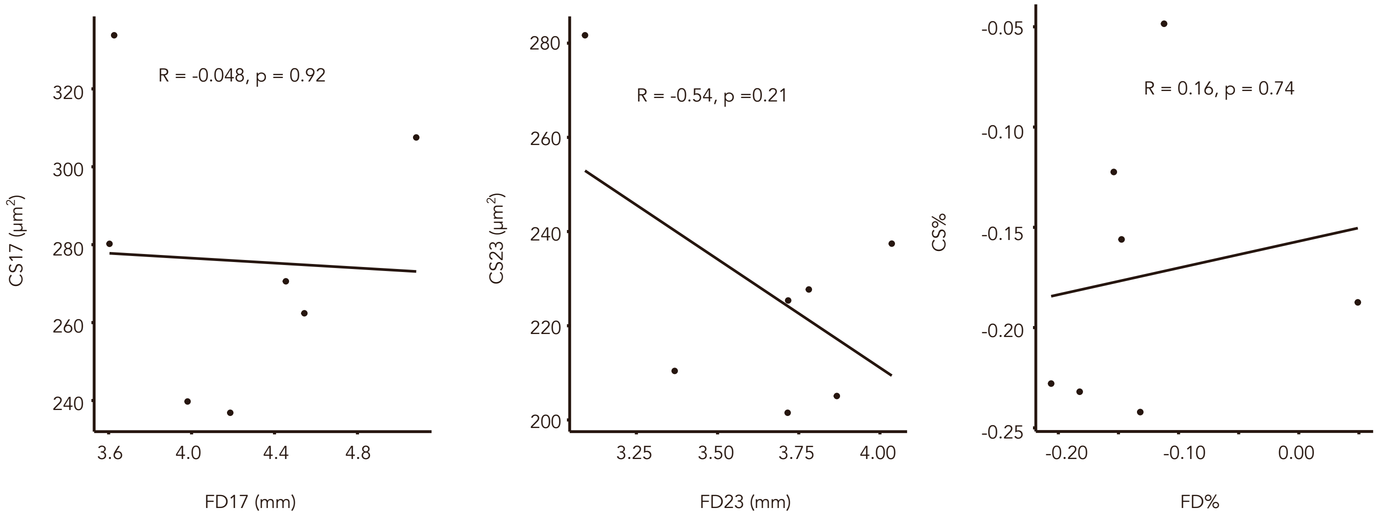

Figure S3. Analysis of meristem and petal size plasticity. Scatter plot of percentage difference between 23°C and 17°C for flower diameter and cell size area.

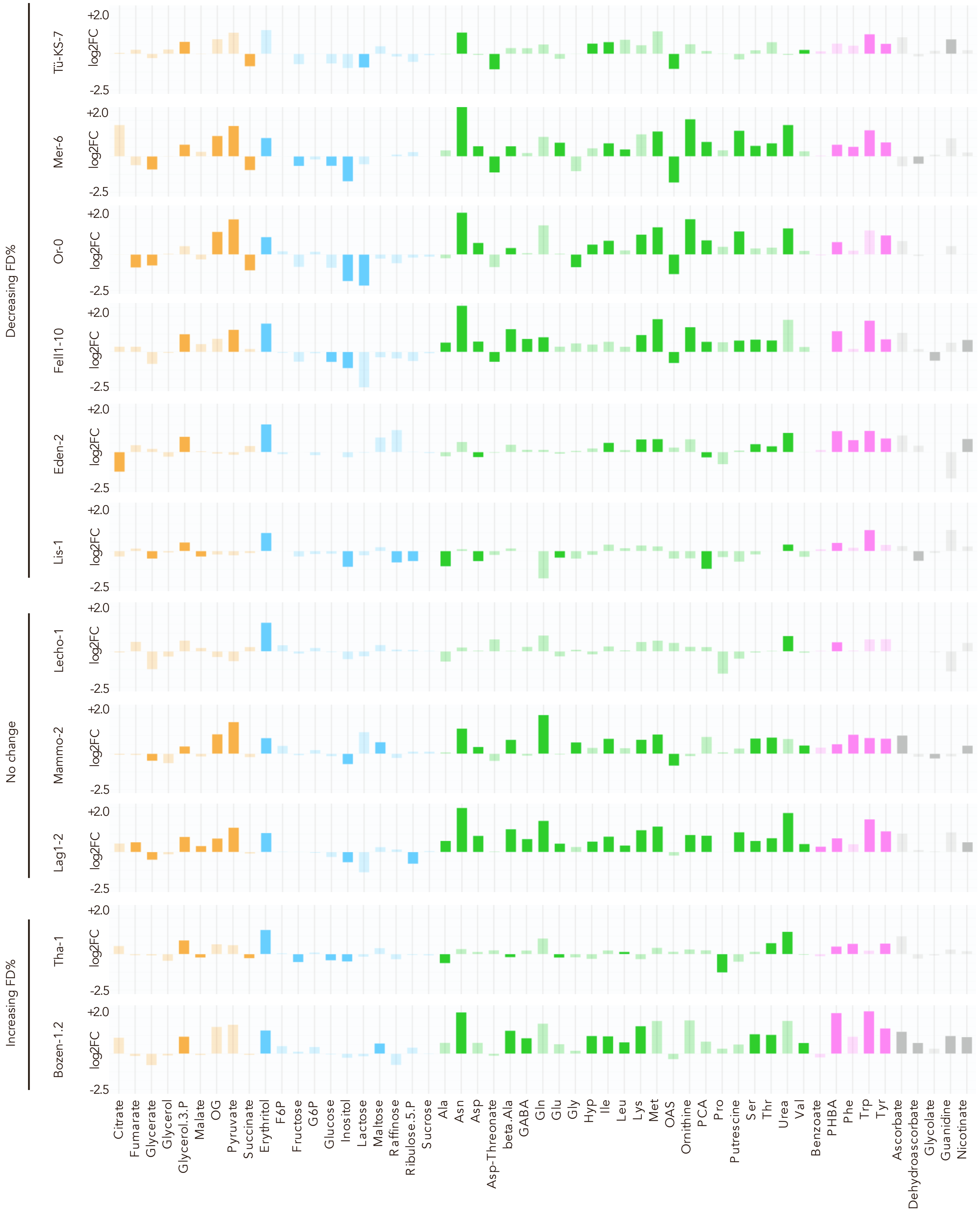

Figure S4A. Content of primary metabolites extracted from flowers of *Arabidopsis thaliana* accessions grown at 17°C and 23°C. Charts show log2 FC of metabolite content normalized by weight and internal standard in flowers grown at 17°C in comparison to 23°C. Metabolites are grouped by colour based on their association with known metabolic pathways of the central metabolism: orange, TCA cycle; blue, sugar and starch metabolism; green, synthesis of amino acids and related metabolites; pink, shikimate pathway; grey, others. Dark coloured bars represent statistically significant changes (t-test, p-value < 0.05). Abbreviations: OG, 2-oxo-glutarate; F6P, fructose-6P; G6P, glucose-6P; OAS, O-acetylserine; PCA, Pyroglutamic acid; PHBA, 4-Hydroxybenzoic.

A

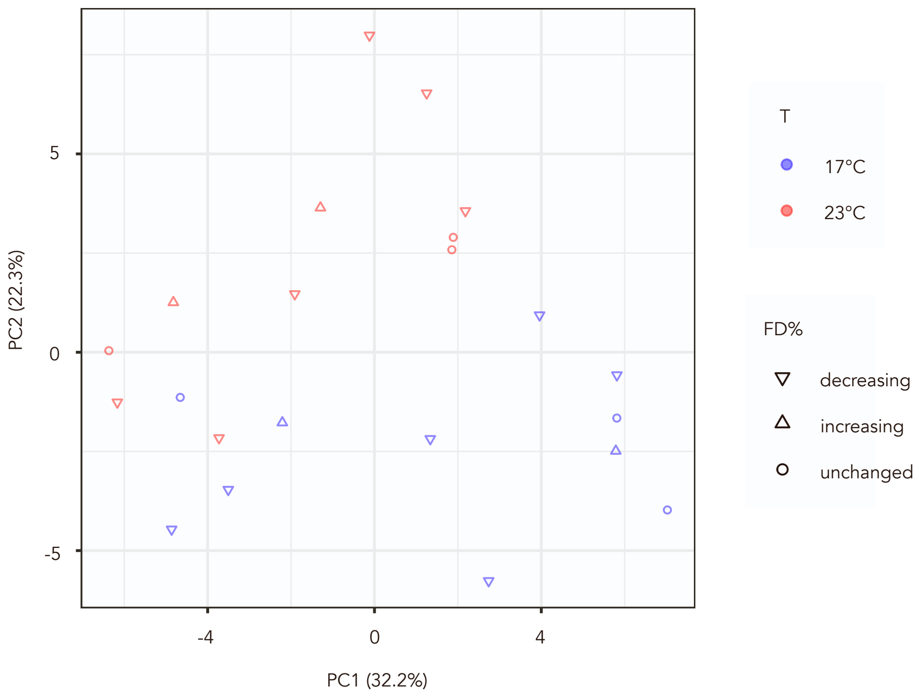

B

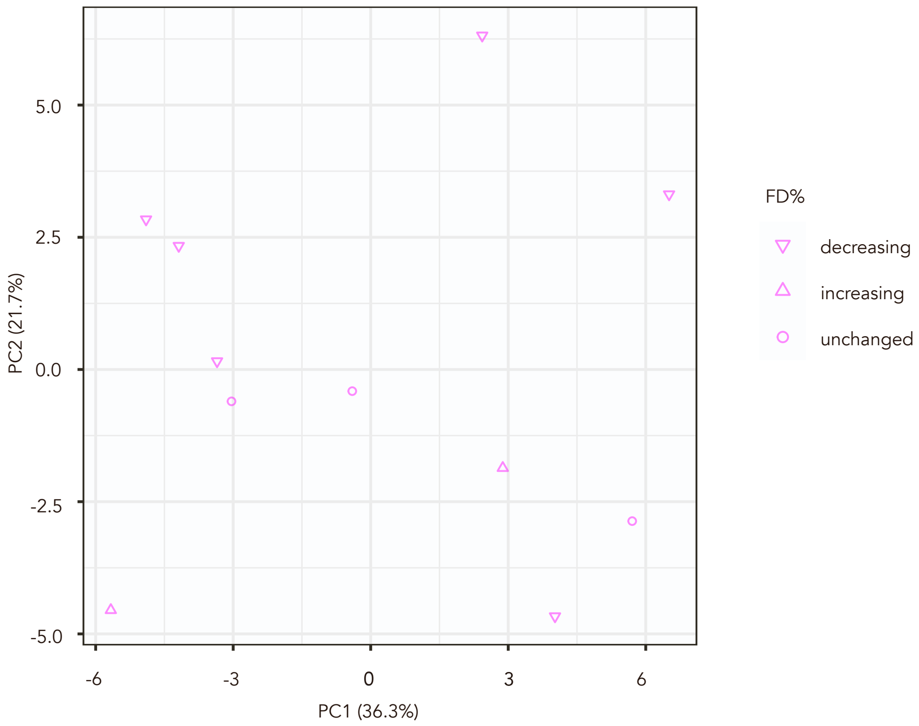

Figure S4B. PCA of primary metabolites in 11 Arabidopsis accessions grown at 17°C and 23°C. Accessions were assigned according to whether they had decreasing FD% (Tü-KS-7, Mer-6, Or-0, Fell1-10, Eden-2 and Lis-1), unchanged (Lag1-2, Mammo-2 and Lecho-1) or increasing (Bozen1-2 and Tha-1). PCA was computed for (A) Log transformed mean metabolite intensity and (B) log2 fold-change of mean metabolite intensity at 23°C compared to 17°C.

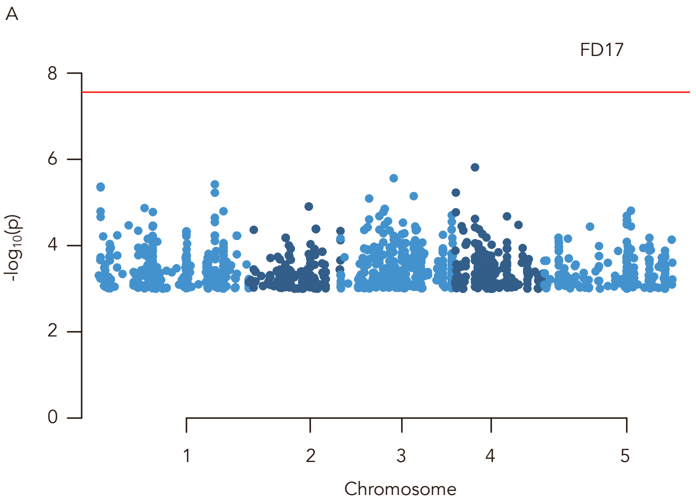

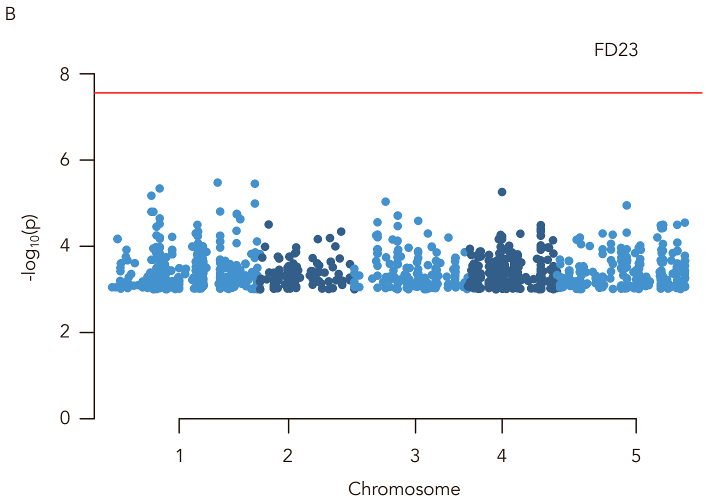

Figure S5. Manhattan plots of significant SNPs in genomic wide association analysis for log10 transformed mean FD at 17°C (A) and 23°C (B). A cutoff of $p < 0.001$ is applied to the displayed SNPSs.

Supplementary table 1. FD data for wild *A. thaliana* and *A. arenosa* plants, related to figure 1

**Species**	**Plant of origin**	**Flower diameter (mm) mean at 17 °C**	**Flower diameter (mm) standard deviation at 17 °C**	**Flower diameter (mm) mean at 23 °C**	**Flower diameter (mm) standard deviation at 23 °C**	**Flower diameter plasticity (%) to temperature ((23 °C - 17 °C)/17 °C)**
A. thaliana	1	5,81	0,43	4,01	0,35	-0,31
A. thaliana	2	5,73	0,56	4,53	0,45	-0,21
A. thaliana	3	5,67	0,55	3,93	0,45	-0,31
A. thaliana	4	4,92	0,52	4,51	0,36	-0,08
A. thaliana	5	5,40	0,60	4,35	0,43	-0,20
A. thaliana	6	5,32	0,38	4,58	0,41	-0,14
A. thaliana	7	4,96	0,62	4,57	0,44	-0,08
A. thaliana	8	4,79	0,53	3,90	0,34	-0,19
A. thaliana	9	5,61	0,71	3,97	0,37	-0,29
A. thaliana	10	4,86	0,66	3,93	0,28	-0,19
A. thaliana	11	5,65	0,67	4,08	0,43	-0,28
A. thaliana	12	5,58	0,46	4,14	0,49	-0,26
A. arenosa	13	13,98	0,85	11,59	0,71	-0,17
A. arenosa	14	13,35	1,36	11,85	0,81	-0,11
A. arenosa	15	14,20	0,77	11,30	1,29	-0,20
A. arenosa	16	13,26	1,36	11,22	0,49	-0,15
A. arenosa	17	12,95	0,93	11,17	0,56	-0,14
A. arenosa	18	13,29	0,62	10,94	0,41	-0,18
A. arenosa	19	11,50	0,92	10,28	0,55	-0,11
A. arenosa	20	12,81	0,99	11,83	0,51	-0,08
A. arenosa	21	13,34	0,63	11,20	1,15	-0,16
A. arenosa	22	12,70	1,05	10,42	0,64	-0,18
A. arenosa	23	14,52	1,06	11,84	0,90	-0,18
A. arenosa	24	12,95	0,78	10,95	0,74	-0,15

Supplementary table 3. Candidate genes proximal to the two most significant GWA identified SNPs, related to figure 2

AGI	Description	Expression
**Chr5:25972950**		
At5g64980	Transcription factor	axis of inflorescence
At5g64990	RAB GTPase homolog H1A	flowers, roots
At5g65000	Repressor of cytokinin deficiency 1 (ROCK1)	petals, carpel, flower
At5g65005	Polynucleotidyl transferase, ribonuclease H-like superfamily protei	low expression
At5g65010	Asparagine Synthetase 2 (ASN2)	rosette, hypcotyl
At5g65015	pre-tRNA	hypcotyl
At5g65020	ANNAT2, ANNEXIN 2, ATANN2	flowers, roots
At5g65030	nitric oxide synthase-interacting protein	seeds, cotyledon
At5g65040	Increased Resistance to Myzus Persicae 1 (IRM1)	stems
At5g65050	Agamous-like 31 (AGL31) /MADS affecting flowering 2 (MAF2)	flowers
At5g65060	Agamous-like 70 (AGL70) /MADS affecting flowering 3 (MAF3)	flowers
At5g65070	Agamous-like 69 (AGL69) / MADS affecting flowering 4 (MAF4)	seeds
At5g65080	Agamous-like 68 (AGL68) / MADS affecting flowering 4 (MAF5)	low expression
**Chr5:21719995**		
At5g53480	Homolog of human KPNB1 (ATKPNB1)	leaves, flowers
At5g53486	Transmembrane protein	low expression
At5g53487	pre-tRNA	SAM
At5g53490	Thylakoid lumenal 17.4 kDa protein (TL17) TPR-like	leaf and cotyledon
At5g53500	Transducin/WD40 repeat-like superfamily protein	leaf and cotyledon
At5g53510	Oligopeptide Transporter (ATOPT9)	pollen
At5g53460	NADH-dependent Glutamate Synthase 1 (GTL1)	root

Table S8. Ridge regression derived coefficients for metabolite contribution to FD17, FD23 and FD plasticity, related to supplemental figure 5.

Components	FD17	FD23	FD%
(Intercept)	-25,4474	-9,8795	-0,1054
Ala	0,0248	-0,0458	0,0042
Ascorbate	-0,0049	0,1086	-0,0040
Asn	0,0564	-0,0156	-0,0011
Asp	0,0726	0,0158	0,0157
Asp.Threonate	0,2476	0,0379	0,0271
Benzoate	-0,1956	0,0985	-0,0152
beta.Ala	-0,0001	0,0275	0,0013
Citrate	0,1005	-0,0388	0,0038
Dehydroascorbate	0,1284	0,0737	-0,0218
Erythritol	0,3540	0,1417	-0,0133
F6P	0,2418	0,1445	0,0544
Fructose	0,0832	0,0080	0,0431
Fumarate	-0,0330	-0,0792	0,0109
G6P	0,1673	0,1265	0,0797
GABA	0,0416	0,1145	0,0413
Galactinol	0,0058	0,0018	-0,0010
Gln	-0,0187	0,0049	0,0042
Glu	0,1373	0,0459	0,0021
Glucose	-0,3205	-0,0407	0,0132
Gly	-0,0700	-0,1286	0,0068
Glycerate	0,1054	0,1044	0,0100
Glycerol	-1,0020	-0,3749	-0,1037
Glycerol.3.P	0,2095	0,1802	0,0488
Glycolate	0,6125	-0,3981	0,0492
Guanidine	0,2152	0,0413	0,0186
Hyp	-0,0769	-0,0452	-0,0315
Ile	0,0339	0,1509	-0,0054
Inositol	0,0261	-0,0002	0,0544
Lactose	0,1522	0,0597	0,0058
Lactulose	0,0867	-0,0151	0,0031
Leu	0,0821	0,0910	0,0263
Lys	0,2460	0,0374	-0,0052
Malate	-0,0876	0,0282	-0,0185
Maltose	0,4587	0,3845	0,0127
Met	-0,0186	-0,0236	-0,0043
Nicotinate	-0,3037	0,0342	-0,0143
OAS	0,6804	0,0867	0,0112
OG	0,0682	-0,1175	0,0084
Ornithine	0,1938	0,0086	-0,0038
PCA	0,0393	0,0026	0,0035
PHBA	-0,0389	0,2540	0,0011
Phe	0,3959	0,1549	0,0456
Pro	-0,0015	0,0521	-0,0454
Putrescine	0,1146	0,0292	-0,0093
Pyruvate	0,1721	-0,0663	0,0002

Raffinose	0,1384	0,0511	-0,0366
Ribulose.5.P	0,0964	0,0548	-0,0052
Ser	-0,0117	0,0171	0,0001
Succinate	0,0783	0,0023	-0,0133
Sucrose	0,2870	0,1789	-0,0127
Thr	-0,0864	-0,1186	0,0358
Trp	-0,0885	0,1088	-0,0007
Tyr	0,0305	0,1944	0,0112
Urea	0,2143	-0,0026	-0,0010
Val	0,0367	-0,0121	0,0255
